# Predicting overdose among individuals prescribed opioids using routinely collected healthcare utilization data

**DOI:** 10.1371/journal.pone.0241083

**Published:** 2020-10-20

**Authors:** Jenny W. Sun, Jessica M. Franklin, Kathryn Rough, Rishi J. Desai, Sonia Hernández-Díaz, Krista F. Huybrechts, Brian T. Bateman

**Affiliations:** 1 Division of Pharmacoepidemiology and Pharmacoeconomics, Department of Medicine, Brigham and Women’s Hospital and Harvard Medical School, Boston, MA, United States of America; 2 Department of Epidemiology, Harvard T. H. Chan School of Public Health, Boston, MA, United States of America; 3 Department of Anesthesiology, Perioperative, and Pain Medicine, Brigham and Women’s Hospital and Harvard Medical School, Boston, MA, United States of America; University of South Australia, AUSTRALIA

## Abstract

**Introduction:**

With increasing rates of opioid overdoses in the US, a surveillance tool to identify high-risk patients may help facilitate early intervention.

**Objective:**

To develop an algorithm to predict overdose using routinely-collected healthcare databases.

**Methods:**

Within a US commercial claims database (2011–2015), patients with ≥1 opioid prescription were identified. Patients were randomly allocated into the training (50%), validation (25%), or test set (25%). For each month of follow-up, pooled logistic regression was used to predict the odds of incident overdose in the next month based on patient history from the preceding 3–6 months (time-updated), using elastic net for variable selection. As secondary analyses, we explored whether using simpler models (few predictors, baseline only) or different analytic methods (random forest, traditional regression) influenced performance.

**Results:**

We identified 5,293,880 individuals prescribed opioids; 2,682 patients (0.05%) had an overdose during follow-up (mean: 17.1 months). On average, patients who overdosed were younger and had more diagnoses and prescriptions. The elastic net model achieved good performance (c-statistic 0.887, 95% CI 0.872–0.902; sensitivity 80.2, specificity 80.1, PPV 0.21, NPV 99.9 at optimal cutpoint). It outperformed simpler models based on few predictors (c-statistic 0.825, 95% CI 0.808–0.843) and baseline predictors only (c-statistic 0.806, 95% CI 0.787–0.26). Different analytic techniques did not substantially influence performance. In the final algorithm based on elastic net, the strongest predictors were age 18–25 years (OR: 2.21), prior suicide attempt (OR: 3.68), opioid dependence (OR: 3.14).

**Conclusions:**

We demonstrate that sophisticated algorithms using healthcare databases can be predictive of overdose, creating opportunities for active monitoring and early intervention.

## Introduction

Over the past two decades, the abuse, dependence, and misuse of prescription opioids has become one of the most widely recognized public health problems in the United States [1–4]. Since 2000, the rate of overdose deaths involving opioids has tripled, with over 70,000 deaths in 2017 and an accumulation of over 700,000 deaths to date [5–9]. The rate of overdose deaths involving prescription opioids is now five times higher than it was in 1999, making it a leading cause of injury-related death in the United States [10–12]. In recent years, the combined economic burden of the opioid epidemic has cost the United States over $50 billion annually [13–16].

Routinely collected healthcare databases, which provide a rich source of longitudinal patient information on medical diagnoses and procedures, medication prescriptions, and healthcare utilization [17] could be leveraged as a resource for surveilling and intervening on patients at high-risk of aberrant opioid-related behaviors. Several automated algorithms to detect opioid-related adverse events have been proposed [18, 19], including two algorithms developed to predict overdose [20, 21]. Such claims-based algorithms have already been implemented in practice as tools for routine surveillance. Examples include the Centers for Medicare and Medicaid Services’ Overutilization Monitoring System in Medicare Part D to help prevent overutilization of prescription opioid medications [22] and a private company’s platform that has licensed its algorithm to organizations for use in identifying patients at risk opioid overdose [23]. Because overdose is such a potentially catastrophic outcome and there are low cost interventions that can be directed to at-risk patients (e.g., naloxone), even algorithms with modest performance may have clinical utility in flagging at-risk patients for intervention.

Previous studies have highlighted that the performance of existing opioid-related algorithms could be improved [18, 24]. Most algorithms currently used in practice are based on simple models with few predictors and have not fully taken advantage of the rich data available in healthcare databases. Recently, one study found that machine learning algorithms based on claims data performed well for risk prediction of opioid overdose in Medicare patients (which insures elderly patients in the United States) [20]. However, recent reports suggest that over 90% of opioid overdoses occur in patients <65 years old [5, 25], and the performance of more sophisticated algorithms based on data-driven techniques has not been evaluated in younger patients. Additionally, machine learning methods for predicting opioid overdose have not been directly compared to traditional multivariate regression.

In a nationwide healthcare database of commercially-insured patients, we used a data-driven approach to develop an algorithm to identify patients prescribed opioids who may be at high-risk of overdose. Specifically, we were interested in developing an approach that could use routinely collected healthcare utilization data to identify high-risk patients who have received prescription opioids and may benefit from interventions that can prevent overdose such as naloxone, a potentially life-saving medication that can be administered to patients suspected to have an overdose [26], or medication-assisted treatment (methadone, buprenorphine, or naltrexone). Such evidence-based practices can be effective in reducing the risk of overdose and will play an important role in preventing future overdoses [27]. To develop this tool, we applied two data-driven approaches and compared their performance to traditional multivariate regression. First, we utilized elastic net penalized regression to empirically select strong predictors of opioid overdose [28]. Then, we evaluated whether random forest, a machine learning method that also automates the identification of interactions between predictors or nonlinear associations between predictors and the outcome, could enhance prediction [29, 30]. To fully take advantage of the information available in the database, we produced a time-updating algorithm. Each month, the patient’s recent medical history was re-assessed, allowing us to capture temporal changes in clinically important risk factors and emulate real-time safety surveillance.

## Materials and methods

### Study population

This study used data from the Optum^©^ Clinformatics® Data Mart, which comprises de-identified US healthcare claims for beneficiaries of a large, national commercial insurance provider. At any given time, Optum covers approximately 13 million people in the United States and reflects a geographically diverse population with beneficiaries from several health plans that have different benefit structures. The database contains individual-level information on inpatient and outpatient diagnoses and procedures, as well as records of outpatient prescription dispensing. Data from October 2011 to September 2015 were used in the analysis.

We identified a cohort of patients at least 18 years old who filled at least 1 prescription of the following opioids: buprenorphine, butorphanol, codeine, fentanyl, hydrocodone, hydromorphone, levorphanol, methadone, meperidine, morphine, oxycodone, oxymorphone, pentazocine, tapentadol, and tramadol. Both incident and prevalent users were eligible for inclusion. The date of the first observed dispensing of any prescription opioid was defined as the index date. Patients with a cancer diagnosis or overdose at any point prior to the index date were excluded. Patients with a prior overdose were excluded because they are at high-risk for recurrent overdose and prescription of naloxone or other interventions are clearly indicated. In our approach, we were interested in identifying patients who have not yet overdosed but could potentially benefit from preventative interventions. To develop our prediction model, we split the sample into 3 datasets. Patients were randomly allocated into the training set (50% of cohort), validation set (25%), or test set (25%).

### Outcome and candidate predictors

We followed patients until first opioid overdose, which was defined as the presence of an inpatient or outpatient diagnosis code for prescription opioid poisoning (*International Classification of Diseases*, *Ninth Revision*, *Clinical Modification* [ICD-9-CM] codes 965.00, 965.02, 965.09) or heroin poisoning (ICD-9-CM code 965.01). A previous validation study showed that these ICD-9-CM diagnosis codes for opioid overdoses and poisonings accurately identify opioid overdose events reported in medical records (PPV = 81–84%) [31]. Patients were censored at the end of insurance enrollment, death, cancer diagnosis, or end of follow-up (September 30, 2015).

**Fig 1:**
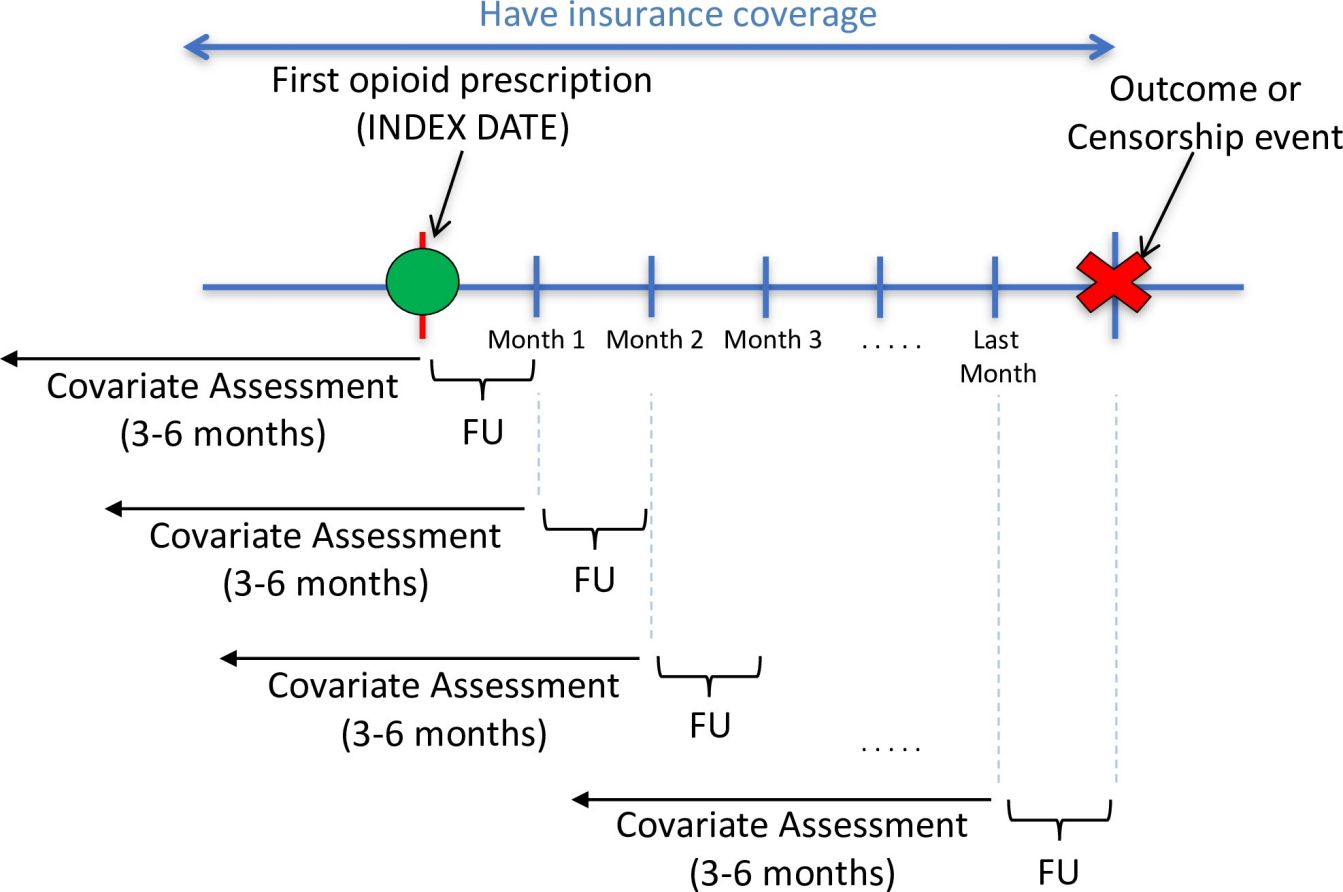
Study design diagram. Abbreviations: FU = follow up. The covariate assessment period was 3 months for medication dispensings and healthcare utilization and 6 months for medical diagnoses.

Seventy-eight candidate predictors were selected *a-priori* based on subject matter knowledge. We considered variables related to demographics, medical diagnoses, medication prescriptions, and healthcare utilization. The ICD-9-CM codes (inpatient or outpatient, any position) used to define the medical diagnoses are displayed in **S1 Table**. All time-varying predictors were updated monthly. Demographic variables were captured on the index date and modeled categorically. Medical diagnoses were modelled as binary variables. All other candidate predictors were modelled as continuous variables. For each person-month of follow-up, medical diagnoses were defined during the preceding 6-month period and all other candidate predictors (medication prescriptions, healthcare utilization) were assessed during the preceding 3-month period. A longer covariate assessment period was used for medical diagnoses to allow sufficient time for diagnoses to be captured. However, six months of available data was not required since the goal was to mimic how active surveillance would be conducted in healthcare databases. If less than 3–6 months of data were available, all obtainable information was used. Therefore, each patient’s recent medical history was updated for each month they were enrolled to account for changes in risk factors over time. This study design is summarized in **Fig 1**.

### Statistical analysis

#### Model development

Pooled logistic regression models were used to predict the odds of opioid overdose in the next month, based on patient history from the prior 3–6 months. We used elastic net regularization, which minimizes overfitting through parameter shrinkage and variable selection, to create a parsimonious algorithm [28]. Our candidate model contained all candidate predictors, as well as quadratic transformations of total number, days supplied, and dose for opioid prescriptions. Inclusion of quadratic transformations was determined *a-priori* to accommodate potential non-linear relationships between key candidate predictors and outcome. Continuous variables were standardized to improve optimization and convergence of the models. Extreme outliers (>4 standard deviations of the mean) were imputed as the mean. To account for time, number of months since first observed opioid dispensing was included as a covariate. Models were fit using data exclusively from patients in the training set.

Decisions to optimize model performance were made using patient data in the validation set. Specifically, mean absolute error (MAE) was used for the tuning of λ, which controls the magnitude of regularization (smaller λ value imposes less penalization). The elastic net procedure generated 72 candidate values for λ from the training set. For each potential λ value, MAE was assessed by computing the mean difference between observed and predicted probabilities. The λ value that minimized the MAE was used in the final model. While K-fold cross-validation is typically used to select the optimal tuning parameter, the size of our data prevented use of this procedure (~50 million rows of person-month data in the training set). However, the differences among validation approaches decreases as sample size increases [32].

For the final model, beta coefficients and odds ratios (OR) were reported. 95% confidence intervals (95% CI) were not provided because elastic net regularization does not provide an accurate estimate of precision [33].

#### Internal validation

Model performance was assessed in the test set. Discrimination was evaluated using c-statistics, which can be interpreted as the probability that the model correctly classifies a random patient who experienced an overdose in a given month as higher risk than a random patient who did not overdose in a given month. Model accuracy was evaluated using the Brier score, which calculates the squared differences between the actual outcomes and the model’s predicted probabilities. A lower Brier score suggests better accuracy [34].

Calibration was assessed visually at the person-month level. We compared the mean observed and predicted probabilities in 29 strata: deciles of predicted probability, with the highest decile further split into 20 additional strata based on percentiles. The highest decile of predicted probability included patients at both high and moderate risk, so further stratification allowed closer examination of patients at the highest risk of overdose and ensured comparable risks for patients within the same strata [34]. A perfectly calibrated model would form a diagonal line, suggesting that the observed incidence of the outcome is equal to the predicted risk of the outcome.

Predicted probabilities from the final elastic net model were used to classify patients into high and low risk groups using several potential thresholds, ranging from 0.0015% to 0.15% probability of having an overdose in the next month. Our time-updating approach means that each patient’s risk of overdose may change each month. However, we anticipate that for most clinical applications, interest will be in intervening at the patient level when high-risk individuals are flagged, as opposed to identifying high-risk person-months. In the primary analysis, sensitivity, specificity, positive predictive value (PPV), and negative predictive value (NPV) were computed at the person-level, instead of the person-month level, for each threshold. A person was therefore classified as high-risk if at least 1 follow up month was flagged as high-risk and classified as a true positive if an overdose occurred at any point during follow up. As an additional analysis, we estimated potential classification at the person-month level, where person-months were considered high-risk if that month was flagged and were considered a true positive if an overdose event occurred in the next month. This classification considers risk for each month separately.

#### Subgroup and secondary analyses

To evaluate the robustness of our primary results, the final elastic net model was validated across subgroups of age (18–25, 26–35, 36–50, 50–65, >65 years) and gender (male, female).

As secondary analyses, we evaluated whether use of different analytic approaches may alter performance. First, we used random forest, a non-parametric data-mining technique, to consider prediction models with all possible variable transformations (polynomials, logarithms, etc.) and interaction terms [35]. Briefly, random forest is a supervised classification method that builds many decision trees to predict the outcome. At each split in a tree, a random sample of predictors is chosen. The overall prediction is comprised of the proportion of trees predicting overdose. Then, to consider the potential gain in using data-driven approaches, we used traditional logistic regression. The random forest and traditional regression models were fit in the training set using the same set of candidate predictors as the elastic net model, and performance was evaluated in the test set. The validation set was not used because there were no hyper-parameters that required tuning, as there were in elastic net.

Additionally, we evaluated whether inclusion of many time-updated predictors outperformed simpler models. First, to examine the potential gain in updating predictors over time, we fit a traditional logistic model using baseline predictors only (measured during the period prior to first opioid prescription) to predict overdose at any point during follow up. Then, to assess whether including many predictors improved performance, we fit a traditional logistic model comprised of only the top 10 most important predictors (updated over time). These predictors were identified based on variable importance (largest mean decrease in accuracy) from the random forest model. They include age, gender, region, back and neck pain, opioid dependence, psychosis, depression, anxiety disorder, number of prescribers for non-opioids, and neuropathic pain.

Elastic net and random forest analyses were performed in R version 3.4.3 using the packages “glmnet” version 2.0–16 and “randomforest” version 4.6–14, respectively [36, 37].

## Results

We identified 5,293,880 individuals who were prescribed opioids, of which 2,682 patients (0.05%) had an observed opioid overdose during follow-up (**S1 Fig**). Patients were followed for a total of 99,174,018 person-months (0.003% of person-months with an overdose) and an average of 12.9 months among patients with an overdose and 17.1 months among patients without an overdose.

For each candidate predictor, descriptive statistics are displayed in **Table 1**. Each individual’s characteristics are updated each month, so the statistics shown reflect measurements during the 3–6 month window prior to overdose or a censorship event (final covariate assessment period). Patients who overdosed were younger than those who did not overdose (25.4% in 18–25 years among overdose vs. 12.6% among no overdose), but other demographic characteristics were relatively similar between groups. Compared to those who did not overdose, patients who overdosed were more likely to have at least 1 diagnosis of opioid dependence (16.6% vs. 0.7%) and opioid abuse without dependence (5.4% vs. 0.1%) during the 6 months prior to overdose or censoring. Additionally, compared to those who did not overdose, patients who overdosed had a higher number of total opioid dispensings (mean [sd], 2.42 [2.61] vs. 0.48 [1.11]), total dose for opioid prescriptions in oral morphine equivalents (mean [sd], 763.95 [1753.85] vs. 96.83 [596.06]), number of unique prescribers of opioids (mean [sd], 1.15 [1.11] vs. 0.32 [0.61]), and number of unique pharmacies for opioid dispensings (mean [sd], 1.05 [0.98] vs. 0.30 [0.55]) during the 3 months prior to censoring. Descriptive statistics at the person-month level are shown in **S2 Table**.

**Table 1:** Characteristics during the window prior to censoring for patients who have filled at least 1 opioid prescription between October 2011 to September 2015.

**Candidate Predictors**	**Overdose (n = 2,682)**		**No Overdose (n = 5,291,198)**	
*Categorical variables*	N	%	N	%
Age				
18–25 yrs	681	25.4	665,428	12.6
26–35 yrs	411	15.3	1,125,043	21.3
36–50 yrs	804	30.0	1,805,509	34.1
51–65 yrs	708	26.4	1,530,798	28.9
>65 yrs	78	2.9	164,420	3.1
Gender				
Female	1488	55.5	2,915,392	55.1
Male	1192	44.4	2,375,409	44.9
Undefined	2	0.1	397	0.0
Geographic Region				
Northeast	204	7.6	409,205	7.7
Midwest	739	27.5	1,329,590	25.1
South	1222	45.5	2,603,343	49.2
West	516	19.2	946,587	17.9
Unknown	1	0.0	2,473	0.0
Opioid dependence	444	16.6	36,230	0.7
Opioid abuse without dependence	145	5.4	4,273	0.1
Back and neck pain	1,321	49.3	1,057,841	20.0
Neuropathic pain and fibromyalgia	834	31.1	543,041	10.3
Chronic pancreatitis	28	1.0	4,558	0.1
Sickle cell disease	2	0.0	1,277	0.0
Migraine	233	8.7	149,317	2.8
Other headache syndromes	57	2.1	35,673	0.7
Peripheral neuropathy	7	0.2	5,831	0.1
Abdominal pain	603	22.5	459,929	8.7
Renal calculus	77	2.9	92,200	1.7
Dental pain	26	1.0	25,324	0.5
Other pain^a^	687	25.6	231,167	4.4
Mild and musculoskeletal injury (sprains & strains)	356	13.3	354,030	6.7
Severe musculoskeletal injury (dislocations, tears, ruptures)	120	4.5	147,708	2.8
Fractures	202	7.5	128,293	2.4
Marijuana use	99	3.7	10,295	0.2
Cocaine use	72	2.7	3,467	0.1
**Candidate Predictors**	**Overdose (n = 2,682)**		**No Overdose (n = 5,291,363)**	
*Categorical variables*	N	%	N	%
Alcohol abuse	283	10.6	46,057	0.9
Tobacco use	549	20.5	288,261	5.5
Other substance use^b^	422	15.7	28,554	0.5
ADHD	151	5.6	118,477	2.2
Depression	1,005	37.5	429,718	8.1
Bipolar disorder	286	10.7	56,286	1.1
Psychosis/schizophrenia	788	29.4	211,477	4.0
Personality disorder	51	1.9	7,034	0.1
Anxiety disorder	849	31.7	407,246	7.7
Other psychiatric disorders ^c^	138	5.2	9,209	0.2
Suicide attempt	84	3.1	1,881	0.0
Hepatic disease	191	7.1	134,306	2.5
Renal insufficiency	24	0.9	9,185	0.2
Endocarditis	7	0.3	893	0.0
HIV	20	0.8	14,398	0.3
*Continuous Variables*	Mean	SD	Mean	SD
Total opioid dispensings	2.42	2.61	0.48	1.11
Number of extended-release opioid prescriptions dispensed	0.46	1.07	0.03	0.29
Total number of days supplied for all opioid prescriptions dispensed	46.91	58.67	7.22	22.76
Total dose (in oral morphine equivalents) for opioid prescriptions dispensed	763.95	1753.85	96.83	596.06
Number of unique prescribers of opioids	1.15	1.11	0.32	0.61
Number of unique pharmacies for opioid dispensings	1.05	0.98	0.30	0.55
Total number of non-opioid prescriptions dispensed	11.38	10.74	4.36	5.75
Number of unique non-opioid generics dispensed	9.84	9.31	3.82	5.06
Number of unique prescribers for non-opioid medications	2.69	2.18	1.34	1.35
Number of unique pharmacies for non-opioid medications	1.73	1.27	0.97	0.88
Number of outpatient visits	10.64	14.07	3.61	14.07
Number of emergency department visits	0.14	0.81	0.09	0.81
Number of hospitalizations	0.27	0.69	0.03	0.69
**Candidate Predictors**	**Overdose (n = 2,682)**		**No Overdose (n = 5,291,363)**	
*Continuous Variables*	Mean	SD	Mean	SD
Number of unique providers seen	6.15	6.47	2.47	3.28
Number of urine drug screens	0.18	1.37	0.00	0.14
Number of opioid dispensings				
Buprenorphine	0.13	0.61	0.01	0.23
Butorphanol	0.00	0.12	0.00	0.06
Codeine	0.00	0.09	0.00	0.06
Fentanyl	0.12	0.65	0.01	0.14
Hydrocodone	0.79	1.43	0.23	0.69
Hydromorphone	0.10	0.54	0.01	0.12
Levorphanol	0.00	0.08	0.00	0.01
Meperidine	0.01	0.12	0.00	0.04
Methadone	0.06	0.41	0.00	0.11
Morphine	0.16	0.71	0.01	0.18
Oxycodone	0.83	1.67	0.10	0.53
Oxymorphone	0.04	0.40	0.00	0.09
Pentazocine	0.00	0.10	0.00	0.03
Tapentadol	0.02	0.21	0.00	0.08
Tramadol	0.23	0.77	0.07	0.40
Number of non-opioid dispensings				
Antidepressants	1.30	1.87	0.35	0.98
Antipsychotics	0.27	0.85	0.02	0.26
Barbituates	0.01	0.12	0.00	0.09
Benzodiazepines	1.14	1.66	0.17	0.64
CNS stimulants	0.13	0.59	0.06	0.42
Gabapentanoids	0.44	1.04	0.06	0.36
Mood stabilizers	0.25	0.82	0.05	0.37
Muscle relaxants	0.58	1.18	0.10	0.44
NSAIDs	0.31	0.76	0.15	0.51
Other hypnotics	0.40	1.07	0.08	0.43
Triptans	0.10	0.76	0.03	0.39

Window prior to censoring (opioid overdose or censorship event) was 6 months for time-varying categorical variables and 3 months for continuous variables.

^a^Other pain: Pain not elsewhere classified, generalized pain, pain disorders related to psychological factors.

^b^Other substance use: Dependence on or non-dependent abuse of sedatives, psychostimulants, hallucinogens, other drugs, or combinations of drugs; Drug dependence complicating pregnancy.

^c^Other psychiatric disorders: Dissociative disorders, neurasthenia, depersonalization disorder, hypochondriasis, somatoform disorders, unspecified nonpsychotic mental disorder, overanxious disorder.

The elastic net model had strong discrimination, with a c-statistic of 0.888 (95% CI: 0.872–0.902), and good accuracy (Brier score: 2.662 x 10^−5^; **Table 2**). Performance of the elastic net model was largely consistent across age and gender subgroups. Using different analytic approaches had little influence on model discrimination (traditional logistic regression c-statistic: 0.881, 95% CI: 0.866–0.896; random forest c-statistic: 0.862, 95% CI: 0.845–0.878). However, simpler models based on baseline predictors only (c-statistic: 0.806, 95% CI: 0.787–0.826) and the top 10 predictors only (c-statistic: 0.825, 95% CI: 0.808–0.843) had weaker performance.

**Table 2:** Comparative performance of models and validation in subgroups in the test set.

Model	c-statistic (95% CI)	Brier Score
*Comparison of Models*		
Traditional logistic regression 1: Baseline only	0.806 (0.787–0.826)	49.740 x 10^−5^
Traditional logistic regression 2: Top 10 predictors only (time-updated)^a^	0.825 (0.808–0.843)	2.641 x 10^−5^
Traditional logistic regression 3: All baseline and time-updating	0.881 (0.866–0.896)	2.671 x 10^−5^
Elastic net (baseline and time-updating)–*Primary Analysis*	0.887 (0.872–0.902)^b^	2.662 x 10^−5^
Random forest (baseline and time-updating)	0.862 (0.845–0.878)	2.640 x 10^−5^ ^b^
*Validation in Subgroups–Elastic net model*		
Age 18–25 years	0.849 (0.808–0.890)	5.304 x 10^−5^
Age 26–35 years	0.861 (0.816–0.907)	2.154 x 10^−5^
Age 36–50 years	0.877 (0.847–0.906)	2.393 x 10^−5^
Age 50–65 years	0.912 (0.890–0.935)	2.355 x 10^−5^
Age >65 years	0.934 (0.901–0.967)	1.820 x 10^−5^
Males	0.882 (0.857–0.907)	2.617 x 10^−5^
Females	0.891 (0.873–0.909)	2.699 x 10^−5^

^a^Top 10 predictors include age, gender, region, back and neck pain, opioid dependence, psychosis, depression, anxiety disorder, number of prescribers for non-opioids, and neuropathic pain. The 10 most important variables were identified based on variable importance (largest mean decrease in accuracy) from the random forest model.

^b^In comparison of models, indicates best performance with respect to the metric (lowest Brier score, highest c-statistic).

**Fig 2:**
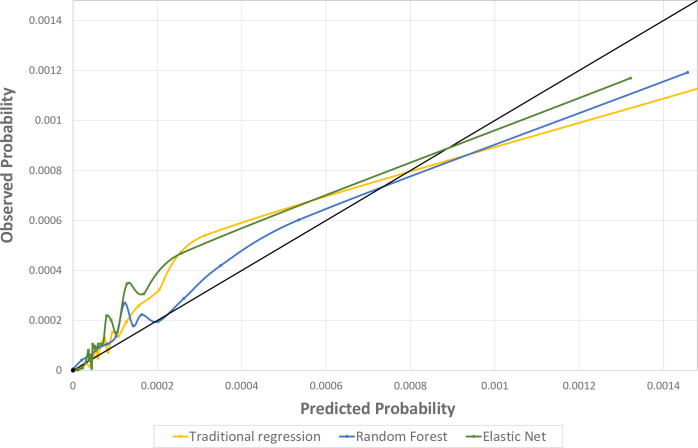
Calibration plot for models predicting opioid overdose in the next month: Comparison of analytic approaches. All models based on baseline and time-updated predictors.

The elastic net model’s predicted probability of opioid overdose in the next month provided close, but slightly underestimated predictions of the observed risk (**Fig 2**). However, the predicted probabilities were higher than the true risk of overdose in patients in the highest percentile of risk (>99.99^th^ percentile). Random forest was slightly better calibrated compared to elastic net and traditional regression, particularly for patients with the highest predicted probabilities of opioid overdose (**Fig 2**).

The final elastic net model identified 40 predictors for opioid overdose in the next month based on the previous 3–6 months of medical history (**Table 3**). All identified predictors were associated with increased odds of opioid overdose. Based on model coefficients, the strongest predictors for opioid overdose were age 18–25 years old at first opioid prescription (OR = 2.21 compared to age 26+ years) and at least 1 diagnosis of the following conditions during the 6 months prior to overdose: suicide attempt (OR = 3.68), opioid dependence (OR = 3.14), opioid abuse without dependence (OR = 2.63), and other substance use (OR = 2.58). Prescriptions for 6 types of opioids during the 3 months prior to overdose (compared to no prescriptions of the opioid type during the prior 3 months) were identified as predictors of overdose: fentanyl (OR = 1.14), hydrocodone (OR = 1.10), hydromorphone (OR = 1.20), methadone (OR = 1.16), morphine (OR = 1.14), and oxycodone (OR = 1.15). Prescriptions for five non-opioid medications during the 3 months prior to overdose were identified as predictors, including benzodiazepines (OR = 1.18) and gabapentanoids (OR = 1.11). Several indicators of healthcare utilization were also identified as predictors, including number of unique pharmacies for opioid dispensings (OR = 1.18) and number of hospitalizations (OR = 1.16). Elastic net does not provide an accurate estimate of precision [33], so for reference, we provided ORs and 95% CIs for the conventional logistic regression that was estimated prior to implementing elastic net (**Table 3**).

**Table 3:** Model coefficients for the traditional multivariate logistic regression model and the elastic net model.

Predictor	Traditional Multivariate Logistic Regression		Elastic Net
	Odds Ratio	95% Confidence Interval	Odds Ratio^b^
*Categorical variables*			
Age			
18–25 yrs	3.73	3.20, 4.35	2.21
26–35 yrs	1.14	0.96, 1.35	ref
36–50 yrs	ref	ref	ref
51–65 yrs	1.00	0.87, 1.16	ref
>65 yrs	1.09	0.78, 1.53	ref
Gender			
Female	0.86	0.77, 0.97	-
Male	ref	ref	-
Unknown	12.14	1.67, 88.33	-
Geographic Region			
Northeast	1.12	0.91, 1.37	-
Midwest	1.17	1.03, 1.33	-
South	ref	ref	-
West	1.03	0.89, 1.19	-
Unknown	<0.001	<0.001, >999.99	-
Opioid dependence	2.92	2.41, 3.55	3.14
Opioid abuse without dependence	2.49	1.89, 3.30	2.63
Back and neck pain	1.25	1.09, 1.43	1.15
Neuropathic pain and fibromyalgia	1.15	0.99, 1.32	1.07
Chronic pancreatitis	1.36	0.78, 2.37	1.01
Sickle cell disease	1.31	0.19, 9.36	-
Migraine	1.01	0.81, 1.26	-
Other headache syndromes	0.97	0.63, 1.48	-
Peripheral neuropathy	0.72	0.40, 1.29	-
Abdominal pain	1.24	1.07, 1.44	1.04
Renal calculus	0.86	0.62, 1.20	-
Dental pain	0.78	0.44, 1.40	-
Other pain^a^	1.20	1.03, 1.40	1.29
Mild and musculoskeletal injury (sprains & strains)	1.05	0.89, 1.24	-
Severe musculoskeletal injury (dislocations, tears, ruptures)	1.06	0.83, 1.37	-
Fractures	1.27	1.02, 1.57	-
Marijuana use	0.81	0.57, 1.14	-
Cocaine use	1.28	0.86, 1.91	1.16
Alcohol abuse	1.86	1.50, 2.31	1.80
Tobacco use	1.42	1.22, 1.65	1.28
Other substance use^b^	2.37	1.93, 2.91	2.58
ADHD	0.88	0.67, 1.16	-
Depression	1.63	1.39, 1.91	1.49
Bipolar disorder	1.26	1.00, 1.58	1.15
Psychosis/schizophrenia	1.70	1.42, 2.05	1.77
Personality disorder	0.67	0.43, 1.04	-
Anxiety disorder	1.25	1.08, 1.45	1.17
Other psychiatric disorders ^c^	1.36	1.00, 1.84	1.28
Suicide attempt	3.85	2.64, 5.62	3.68
Hepatic disease	1.49	1.19, 1.87	1.15
Renal insufficiency	0.90	0.50, 1.65	-
Endocarditis	0.79	0.18, 3.48	-
HIV	1.36	0.67, 2.76	-
*Continuous Variables*			
Total opioid dispensings	1.13	1.01, 1.27	-
Number of extended-release opioid prescriptions dispensed	1.19	1.09, 1.29	1.10
Total number of days supplied for all opioid prescriptions dispensed, for 30-days supplied	1.38	1.19, 1.58	1.09
Total dose (in oral morphine equivalents) for opioid prescriptions dispensed, per 1,000 MME	1.38	1.23, 1.54	1.11
Number of unique prescribers of opioids	1.10	1.00, 1.20	1.10
Number of unique pharmacies for opioid dispensings	1.14	1.03, 1.26	1.18
Total number of non-opioid prescriptions dispensed	1.03	1.01, 1.06	1.00
Number of unique non-opioid generics dispensed	0.97	0.94, 0.99	-
Number of unique prescribers for non-opioid medications	0.98	0.94, 1.02	-
Number of unique pharmacies for non-opioid medications	1.02	0.96, 1.08	-
Number of outpatient visits	0.99	0.99, 1.00	-
Number of emergency department visits	0.99	0.93, 1.05	-
Number of hospitalizations	1.16	1.07, 1.25	1.16
Number of unique providers seen	1.01	0.99, 1.03	1.01
Number of urine drug screens	1.05	1.02, 1.08	1.04
Number of opioid dispensings			
Buprenorphine	0.89	0.80, 0.98	-
Butorphanol	1.15	0.80, 1.64	-
Codeine	1.07	0.46, 2.49	-
Fentanyl	1.25	1.14, 1.38	1.14
Hydrocodone	1.17	1.11, 1.22	1.10
Hydromorphone	1.25	1.16, 1.35	1.20
Levorphanol	<0.001	<0.001, >999.99	-
Meperidine	1.37	0.85, 2.19	-
Methadone	1.22	1.08, 1.39	1.16
Morphine	1.22	1.12, 1.33	1.14
Oxycodone	1.20	1.15, 1.25	1.15
Oxymorphone	1.08	0.92, 1.27	-
Pentazocine	1.22	0.70, 2.12	-
Tapentadol	1.09	0.88, 1.35	-
Tramadol	0.99	0.90, 1.08	-
Number of non-opioid dispensings			
Antidepressants	1.07	1.03, 1.11	1.04
Antipsychotics	0.98	0.91, 1.05	-
Barbituates	0.82	0.47, 1.43	-
Benzodiazepines	1.19	1.15, 1.23	1.18
CNS stimulants	0.87	0.78, 0.97	-
Gabapentanoids	1.11	1.05, 1.17	-
Mood stabilizers	1.03	0.96, 1.10	1.11
Muscle relaxants	1.10	1.05, 1.16	-
NSAIDs	0.98	0.91, 1.05	1.09
Other hypnotics	1.10	1.04, 1.16	1.05
Triptans	0.97	0.90, 1.06	-
*Quadratic Transformations*			
Total opioid dispensings	0.97	0.96, 0.98	0.99
Total number of days supplied for all opioid prescriptions dispensed, for 30-days supplied	0.96	0.94, 0.97	-
Total dose (in oral morphine equivalents) for opioid prescriptions dispensed, per 1,000 MME	0.97	0.95, 0.98	-

^a^Elastic net regularization does not provide an accurate measure of precision, so only odds ratios were provided. Predictors that were dropped after variable selection are indicated with a “-“.

At the person-level, several cut points based on predicted probabilities could be used to dichotomize patients into high and low risk groups (**Table 4 and S3**). Among all potential cut points our algorithm had a high NPV (99.9% for all cut point), and a low PPV, ranging from 0.06% to 3.66%, which was driven by the very low incidence of opioid overdose. Diagnostics at the person-month level performed similarly, with a high NPV and low PPV (**S3 Table**). However, the PPV was much smaller, ranging from 0.003% to 0.26%, as the risk of opioid overdose in each month (0.003%) is much lower than the risk of overdose at any point during follow up (0.05%).

**Table 4:** Performance of elastic net model predictions for classifying patients into high-risk and low-risk groups.

Performance Metric	Optimal Cut point (Maximizes Sensitivity-Specificity Tradeoff)	Cut point Maximizing Sensitivity	Cut point Maximizing Specificity
Predicted probability cut point (%)	0.004	0.0015	0.55
Patients classified as high-risk (%)	20.0	82.4	0.1
No. (%) events in high-risk group	522 (0.21)	645 (0.06)	33 (3.66)
No. (%) nonevents in high-risk group	252,970 (99.79)	1,090,309 (99.94)	868 (96.33)
Sensitivity (%)	80.2	98.5	5.0
Specificity (%)	80.1	17.6	99.9
PPV (%)	0.21	0.06	3.66
NPV (%)	99.9	99.9	99.9
LR_positive_	4.0	1.2	76.8
LR_negative_	0.3	0.1	0.95

Abbreviations: LR = Likelihood ratio; NPV = Negative predictive value; PPV = Positive predictive value.

## Discussion

Using data-driven methods and a time-updating approach, we developed a prediction model for opioid overdose using data routinely collected in healthcare utilization claims. The final algorithm based on elastic net had strong performance with respect to discrimination and was well calibrated. Use of different analytic techniques (elastic net vs. traditional regression vs. random forest) had a relatively small impact on model performance, whereas inclusion of many time-updating predictors substantially improved prediction in the traditional regression. The final algorithm identified 40 characteristics from a patient’s previous 3–6 month medical history that were predictive of opioid overdose in the next month. These findings suggest that high-dimensional algorithms for opioid overdose based on many time-updated predictors could be used by health systems or payers to monitor patients and help identify those at high-risk for opioid overdose and then target them for interventions, including naloxone prescribing.

Implementation could provide an opportunity for real-time surveillance and early intervention. Details on how to calculate the predicted probability of opioid overdose in the next month for an example patient are shown in **S4 Table**. The predicted probability could be used to determine whether the patient is at high-risk of overdose. This process could be automated and repeated each month to ensure active surveillance. Thus, this algorithm could be used to monitor patients or automate the detection of high-risk individuals, whose risk factors are already being routinely collected. As a result, this may facilitate early intervention to mitigate the risk of overdose through prescription of naloxone or other clinical strategies, such as opioid tapering or substance abuse treatment referral.

We proposed several potential cut points for classifying high-risk patients, allowing those who implement the algorithm to determine the optimal thresholds for their intervention of interest. Patients could have been flagged as high-risk during multiple months. However, clinical intervention happens at the patient level, so we considered patients as high-risk if any of their person-months were flagged as high-risk. The selection of the cut point inevitability results in a tradeoff between sensitivity and specificity. For example, the optimal cut point of 0.004% would provide a sensitivity of 80.2% and specificity of 80.1%., while a cut point of 0.15% would maximize specificity (99.8%) at the expense of decreasing sensitivity (13.1%). Since the incidence of overdose is low, the PPV was relatively low among all potential thresholds (PPV<3.66%). A highly specific cut point could be used to maximize the PPV, although most of the patients flagged as high-risk would not go on to have an observed opioid overdose event. However, given the seriousness of the outcome of overdose and the availability of low-cost and low-risk interventions for overdose, a low PPV may still have clinical utility. Over an average 17-month follow up, the risk of overdose was 0.05% among the general population of patients with at least 1 opioid prescription, which is much lower than the risk of overdose among patients flagged as high-risk by our algorithm (0.20% using the optimal cut point, 3.66% when maximizing specificity).

Our algorithm builds on previously proposed algorithms for identifying patients at high-risk of opioid overdose in claims data [20]. In addition to considering baseline factors, we constructed our algorithm to accommodate a large number of time-updating predictors in a setup that closely resembles active safety surveillance and to provide information on the predicted probability of overdose over time with monthly updates. Including a large number of time-updated predictors enhanced algorithm performance. Further, we utilized machine learning approaches to address the issue of overfitting that is routinely encountered in prediction models. Recently, an algorithm to predict opioid overdose in Medicare patients was published [21]. Using similar methods, this study found that machine learning algorithms performed well with respect to risk prediction and stratification of overdose. We demonstrate that data-driven algorithms using administrative data are predictive of overdose not only in the elderly, publicly-insured population, but also in commercially-insured populations, where this surveillance tool can be applied to a broader range of patients who may be at higher risk of overdose and may have slightly different risk factors for overdose.

Our study has several limitations. First, opioid overdose events may be under-recorded in claims data [38]. We only detect overdoses that result in presentation to an emergency department or inpatient admissions. Fatal opioid overdoses may also be under captured since death is not a billable event, but the proportion of fatal overdoses is likely small relative to nonfatal overdoses [39, 40]. The underestimated incidence of overdose suggests that our model’s PPV may be underestimated. Second, our study focuses on a population who are dispensed prescription opioids. Many individuals may receive opioids from nonmedical settings, such as family and friends [41]. These exposures are not well captured in claims data. Future work will be needed to determine if information available in claims may be useful for evaluating the risk of overdose in patients who use opioids illicitly. Third, our study consisted of patients who received prescription opioids, including buprenorphine or methadone. This study population may have captured different types of patients (e.g., those who are doctor shopping and those who are receiving treatment for opioid use disorder) who may benefit from different interventions. Additionally, the data were left-censored at October 2011. Although we used an all-available lookback window to exclude patients with a prior overdose, do not know whether the patients in our study population had a diagnosed overdose prior to October 2011. Another limitation is that the list of candidate predictors does not encompass all of the important risk factors of overdose, such as behavioral health and criminal justice variables that are poorly captured in claims data [42]. Predictors were also measured during the previous 3–6 months, and we did not assess whether a longer covariate assessment period could have resulted in better prediction. Despite the potential incomplete capture all relevant predictors, we highlight that high-dimensional time-updated algorithms can outperform simpler models based on a few predictors, and such methodology can be extended to other data sources. Future work will need to explore whether future expanding the range of predictors improves model performance. Next, internal validation was used to assess model performance. Generalizability to other populations, such as those insured by Medicaid, will need to be assessed in future studies. Finally, we defined medical conditions using ICD-9-CM codes, but the US recently transitioned to *International Classification of Diseases*, *Tenth Revision*, *Clinical Modification* (ICD-10-CM) [43]. Our goal was to capture the relevant medical conditions, as opposed to the specific codes, that predict overdose. Therefore, our algorithm can be implemented in ICD-10-CM through mapping the ICD-9-CM codes to ICD-10-CM.

In conclusion, this study suggests that sophisticated algorithms using data routinely collected in healthcare utilization claims can be predictive of opioid overdose. It highlights the feasibility of using high-dimensional algorithms by payers to create monitoring programs to prospectively identify high-risk patients and create an opportunity for intervention, such as administering naloxone, before an overdose occurs.

## Supporting information

S1 Fig Flow diagram of cohort assembly. (PDF)

S1 Table ICD-9-CM codes for candidate predictors. (DOCX)

S2 Table Characteristics for each person-month of follow up among patients with ≥1 opioid prescription, October 2011 to September 2015. (DOCX)

S3 Table Diagnostics for dichotomizing into “high” and “low” risk groups using different cutpoints. (DOCX)

S4 Table Example calculation for the predicted probability of opioid overdose in the next month. (DOCX)
