# Supplementary figures and images for: Predicting overdose among individuals prescribed opioids using routinely collected healthcare utilization data
Source: PLoS One. 2020 Oct 20;15(10):e0241083. doi: 10.1371/journal.pone.0241083 (PMC7575098; doi:10.1371/journal.pone.0241083)

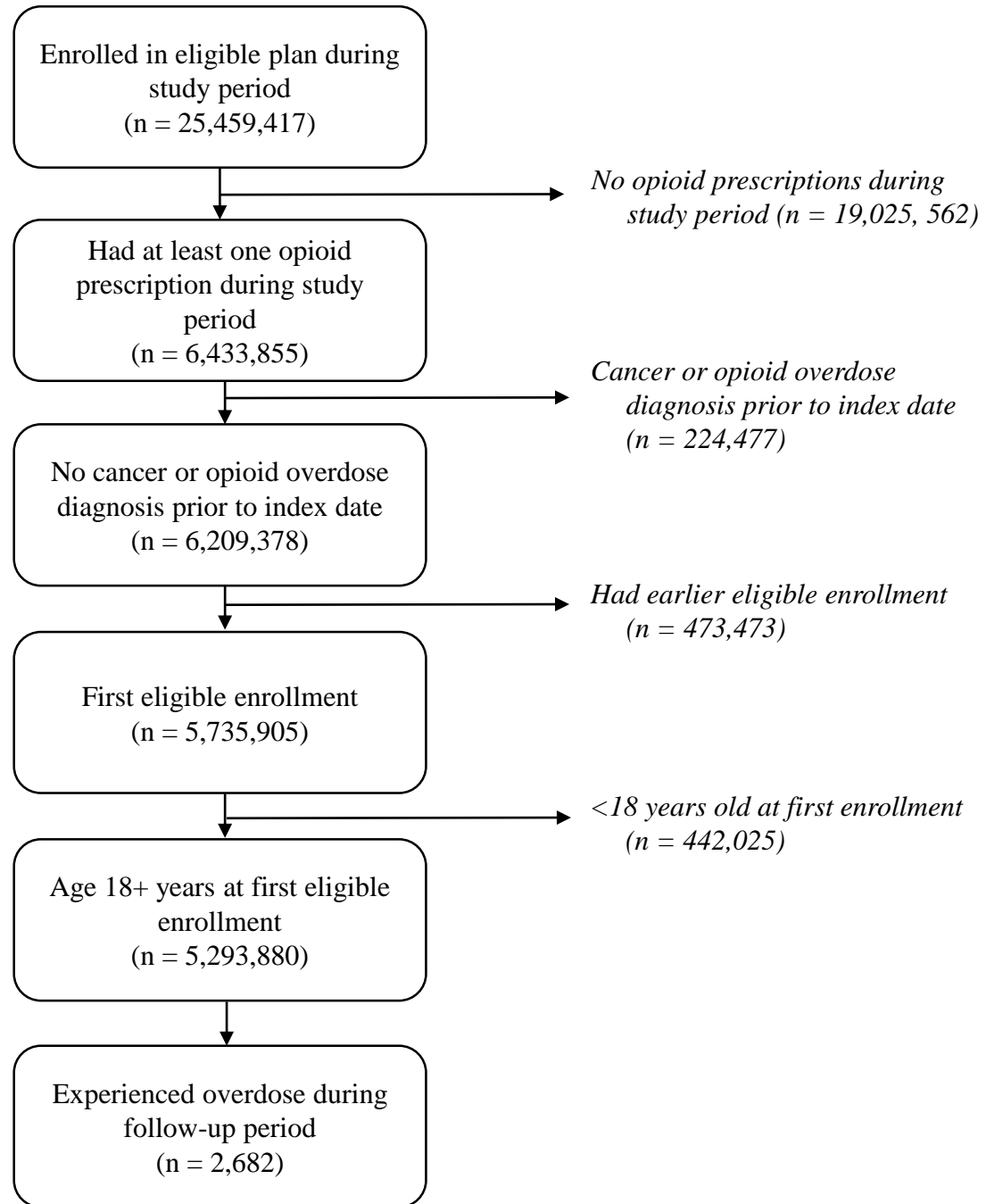

Supplement: S1 Fig — (PDF) [file pone.0241083.s001.pdf]
