# Supplementary material for: Predicting overdose among individuals prescribed opioids using routinely collected healthcare utilization data
Source: PLoS One. 2020 Oct 20;15(10):e0241083. doi: 10.1371/journal.pone.0241083 (PMC7575098; doi:10.1371/journal.pone.0241083)
Supplement: S1 Table — (DOCX) [file pone.0241083.s002.docx]

**S1 Table. ICD-9-CM Codes for Candidate Predictors**

Medical diagnoses were defined as the presence of an inpatient or outpatient ICD-9-CM code in any position.

| **Condition** | **ICD-9 CM Code** |
| --- | --- |
| *Included in final model:* | |
| Opioid dependence | 304.0, 304.00, 304.01, 304.02, 304.7, 304.70, 304.71, 304.72 |
| Opioid abuse without dependence | 305.5, 305.50, 305.51, 305.52 |
| Back and neck pain | 720.xx - 724.xx, 738.4x, 739.1-739.4, 846.xx, 847.xx |
| Neuropathic pain and fibromyalgia | 053.1x, 250.6x, 337.1x, 337.2x, 350.1x, 350.2x, 352.1x, 353.xx-355.xx, 357.xx, 721.1x, 721.41, 721.42, 721.91, 722.7x, 723.4x, 724.4x, 729.1x, 729.2x |
| Chronic pancreatitis | 577.1x |
| Abdominal pain | 789.0x |
| Other pain | 338.xx, 780.96, 307.8x (besides 307.81) |
| Cocaine use | 305.6x, 304.2x, 970.81 |
| Alcohol abuse | 291.xx, 303.0x - 303.9x, 357.5x, 425.5x, 535.30, 535.31, 571.0x - 571.3x, 305.0x, V11.3, E860.0 |
| Tobacco use | 305.1x, 649.0x, 989.84, V15.82 |
| Other substance use | 304.1x, 304.4x-304.6x, 304.8x, 304.9x, 305.3x, 305.4x, 305.7x-305.9x, 648.3x |
| Depression | 293.83, 296.2x, 296.3x, 298.0x, 300.4x, 309.0x, 309.1x, 309.28, 311.xx |
| Bipolar disorder | 296.0x, 296.1x, 296.4x-296.8x, 296.99 |
| Psychosis/schizophrenia | 290.8x, 290.9x, 295.xx, 297.xx-299.xx, 780.1x |
| Anxiety disorder | 293.84, 300.0x, 300.2x, 300.3x, 308.0x, |
| Other psychiatric disorders | 300.1x, 300.5x-300.9x, 309.81, 313.0x |
| Suicide attempt | E95.xx |
| Hepatic disease | 250.4x, 274.10, 403.xx, 404.xx, 440.1x, 442.1x, 453.3x, 572.4x, 580.xx-587.xx, 593.xx, 642.1x, 753.0x, 753.3x, 791.2x, 791.3x, 866.00, 866.01, 866.1x |
| *Not included in final model:* | |
| Sickle cell disease | 282.6x |
| Migraine | 346.xx |
| Other headache syndromes | 307.81, 339.0x - 339.8x |
| Peripheral neuropathy | 356.0x, 356.8x, 337.1x |
| Renal calculus | 592.xx, 594.xx |
| Dental pain | 520.6, 520.8, 520.9, 521.0x, 521.81, 522.xx |
| Mild musculoskeletal injury (sprains & strains) | 840.xx-848.xx |
| Severe musculoskeletal injury (dislocations, tears, ruptures) | 830.xx-839.xx, 717.xx, 718.1x-718.3x, 718.9x, 727.5x-727.6x |
| Fractures | 800.xx-829.xx |
| Marijuana use | 305.2x, 304.3x |
| ADHD | 312.xx, 314.xx |
| Personality disorder | 301.xx |
| Renal insufficiency | 039.1, 042.91, 155.0, 155.1, 155.2, 570.xx, 571.x, 572.x, 573.x, 576.8 |
| Endocarditis | 421.xx |
| HIV | 042.xx, 043.xx, 044.xx, V08.xx, 079.53, 795.71, 795.8x |
