# Supplementary material for: Predicting overdose among individuals prescribed opioids using routinely collected healthcare utilization data
Source: PLoS One. 2020 Oct 20;15(10):e0241083. doi: 10.1371/journal.pone.0241083 (PMC7575098; doi:10.1371/journal.pone.0241083)
Supplement: S2 Table — (DOCX) [file pone.0241083.s003.docx]

**S2 Table. Characteristics for each Person-Month of Follow Up Among Patients with** ≥**1 Opioid Prescription, October 2011 to September 2015**

| **Candidate Predictors** | **Overdose**  **(n=2,682)** | | **No Overdose**  **(n=99,171,336)** | |
| --- | --- | --- | --- | --- |
| *Categorical variables* | N | % | N | % |
| Age |  |  |  |  |
| 18-25 yrs | 681 | 25.4 | 11,796,801 | 11.9 |
| 26-35 yrs | 411 | 15.3 | 19,171,486 | 19.3 |
| 36-50 yrs | 804 | 30.0 | 34,796,470 | 35.1 |
| 51-65 yrs | 708 | 26.4 | 30,148,658 | 30.4 |
| >65 yrs | 78 | 2.9 | 3,257,921 | 3.3 |
| Gender |  |  |  |  |
| Male | 1192 | 44.4 | 44,463,155 | 44.8 |
| Female | 1488 | 55.5 | 54,704,335 | 55.2 |
| Undefined | 2 | 0.1 | 3,846 | 0.0 |
| Geographic Region |  |  |  |  |
| Northeast | 204 | 7.6 | 7,791,055 | 7.9 |
| Midwest | 739 | 27.5 | 25,684,791 | 25.9 |
| South | 1222 | 45.5 | 47,779,551 | 48.2 |
| West | 516 | 19.2 | 17,872,941 | 18.0 |
| Unknown | 1 | 0.0 | 42,998 | 0.0 |
| Opioid dependence | 444 | 16.6 | 598,819 | 0.6 |
| Opioid abuse without dependence | 145 | 5.4 | 61,864 | 0.1 |
| Back and neck pain | 1,321 | 49.3 | 20,933,775 | 21.1 |
| Neuropathic pain and fibromyalgia | 834 | 31.1 | 10,773,737 | 10.9 |
| Chronic pancreatitis | 28 | 1.0 | 80,439 | 0.1 |
| Sickle cell disease | 2 | 0.0 | 23,389 | 0.0 |
| Migraine | 233 | 8.7 | 2,901,249 | 2.9 |
| Other headache syndromes | 57 | 2.1 | 669,357 | 0.7 |
| Peripheral neuropathy | 7 | 0.2 | 105,991 | 0.1 |
| Abdominal pain | 603 | 22.5 | 8,870,204 | 8.9 |
| Renal calculus | 77 | 2.9 | 1,841,718 | 1.9 |
| Dental pain | 26 | 1.0 | 480,857 | 0.5 |
| Other pain^a^ | 687 | 25.6 | 4,147,681 | 4.2 |
| Mild and musculoskeletal injury (sprains & strains) | 356 | 13.3 | 7,290,453 | 7.4 |
| Severe musculoskeletal injury (dislocations, tears, ruptures) | 120 | 4.5 | 3,224,928 | 3.3 |
| Fractures | 202 | 7.5 | 2,585,184 | 2.6 |
| Marijuana use | 99 | 3.7 | 162,243 | 0.2 |
| Cocaine use | 72 | 2.7 | 49,816 | 0.1 |
| **Candidate Predictors** | **Overdose**  **(n=2,682)** | | **No Overdose**  **(n=99,171,336)** | |
| *Categorical variables* | N | % | N | % |
| Alcohol abuse | 283 | 10.6 | 759,225 | 0.8 |
| Tobacco use | 549 | 20.5 | 4,879,967 | 4.9 |
| Other substance use^b^ | 422 | 15.7 | 461,201 | 0.5 |
| ADHD | 151 | 5.6 | 2,057,240 | 2.1 |
| Depression | 1,005 | 37.5 | 7,922,119 | 8.0 |
| Bipolar disorder | 286 | 10.7 | 1,003,181 | 1.0 |
| Psychosis/schizophrenia | 788 | 29.4 | 3,816,069 | 3.9 |
| Personality disorder | 51 | 1.9 | 121,989 | 0.1 |
| Anxiety disorder | 849 | 31.7 | 7,141,750 | 7.2 |
| Other psychiatric disorders^c^ | 138 | 5.2 | 150,752 | 0.2 |
| Suicide attempt | 84 | 3.1 | 27,889 | 0.0 |
| Hepatic disease | 191 | 7.1 | 2,423,789 | 2.4 |
| Renal insufficiency | 24 | 0.9 | 139,439 | 0.1 |
| Endocarditis | 7 | 0.3 | 14,254 | 0.0 |
| HIV | 20 | 0.8 | 256,221 | 0.3 |
|  |  |  |  |  |
| *Continuous Variables* | Mean | SD | Mean | SD |
| Total opioid dispensings | 2.42 | 2.61 | 0.63 | 1.3 |
| Number of extended-release opioid prescriptions dispensed | 0.46 | 1.07 | 0.04 | 0.36 |
| Total number of days supplied for all opioid prescriptions dispensed | 46.91 | 58.67 | 9.03 | 26.97 |
| Total dose (in oral morphine equivalents) for opioid prescriptions dispensed | 763.95 | 1753.85 | 106.48 | 634.08 |
| Number of unique prescribers of opioids | 1.15 | 1.11 | 0.4 | 0.65 |
| Number of unique pharmacies for opioid dispensings | 1.05 | 0.98 | 0.38 | 0.59 |
| Total number of non-opioid prescriptions dispensed | 11.38 | 10.74 | 4.68 | 5.96 |
| Number of unique non-opioid generics dispensed | 9.84 | 9.31 | 4.05 | 5.18 |
| Number of unique prescribers for non-opioid medications | 2.69 | 2.18 | 1.41 | 1.35 |
| Number of unique pharmacies for non-opioid medications | 1.73 | 1.27 | 1.03 | 0.89 |
| Number of outpatient visits | 10.64 | 14.07 | 3.68 | 5.74 |
| Number of emergency department visits | 0.14 | 0.81 | 0.11 | 0.74 |
| Number of hospitalizations | 0.27 | 0.69 | 0.03 | 0.19 |
| **Candidate Predictors** | **Overdose**  **(n=2,682)** | | **No Overdose**  **(n=99,171,336)** | |
| *Continuous Variables* | Mean | SD | Mean | SD |
| Number of unique providers seen | 6.15 | 6.47 | 2.51 | 3.15 |
| Number of urine drug screens | 0.18 | 1.37 | 0.01 | 0.17 |
| Number of opioid dispensings |  |  |  |  |
| Buprenorphine | 0.13 | 0.61 | 0.02 | 0.25 |
| Butorphanol | 0.00 | 0.12 | 0.00 | 0.07 |
| Codeine | 0.00 | 0.09 | 0.00 | 0.07 |
| Fentanyl | 0.12 | 0.65 | 0.01 | 0.16 |
| Hydrocodone | 0.79 | 1.43 | 0.27 | 0.78 |
| Hydromorphone | 0.10 | 0.54 | 0.01 | 0.13 |
| Levorphanol | 0.00 | 0.08 | 0.00 | 0.01 |
| Meperidine | 0.01 | 0.12 | 0.00 | 0.04 |
| Methadone | 0.06 | 0.41 | 0.00 | 0.12 |
| Morphine | 0.16 | 0.71 | 0.01 | 0.20 |
| Oxycodone | 0.83 | 1.67 | 0.11 | 0.58 |
| Oxymorphone | 0.04 | 0.40 | 0.00 | 0.11 |
| Pentazocine | 0.00 | 0.10 | 0.00 | 0.03 |
| Tapentadol | 0.02 | 0.21 | 0.00 | 0.09 |
| Tramadol | 0.23 | 0.77 | 0.08 | 0.44 |
| Number of non-opioid dispensings |  |  |  |  |
| Antidepressants | 1.30 | 1.87 | 0.38 | 1.04 |
| Antipsychotics | 0.27 | 0.85 | 0.03 | 0.28 |
| Barbituates | 0.01 | 0.12 | 0.00 | 0.1 |
| Benzodiazepines | 1.14 | 1.66 | 0.18 | 0.68 |
| CNS stimulants | 0.13 | 0.59 | 0.06 | 0.44 |
| Gabapentanoids | 0.44 | 1.04 | 0.06 | 0.39 |
| Mood stabilizers | 0.25 | 0.82 | 0.05 | 0.39 |
| Muscle relaxants | 0.58 | 1.18 | 0.11 | 0.48 |
| NSAIDs | 0.31 | 0.76 | 0.17 | 0.55 |
| Other hypnotics | 0.40 | 1.07 | 0.09 | 0.47 |
| Triptans | 0.10 | 0.76 | 0.04 | 0.42 |

^a^Other pain: Pain not elsewhere classified, generalized pain, pain disorders related to psychological factors

^b^Other substance use: Dependence on or non-dependent abuse of sedatives, psychostimulants, hallucinogens, other drugs, or combinations of drugs; Drug dependence complicating pregnancy

^c^Other psychiatric disorders: Dissociative disorders, neurasthenia, depersonalization disorder, hypochondriasis, somatoform disorders, unspecified nonpsychotic mental disorder, overanxious disorder
