# Supplementary material for: Predicting overdose among individuals prescribed opioids using routinely collected healthcare utilization data
Source: PLoS One. 2020 Oct 20;15(10):e0241083. doi: 10.1371/journal.pone.0241083 (PMC7575098; doi:10.1371/journal.pone.0241083)
Supplement: S3 Table — (DOCX) [file pone.0241083.s004.docx]

**S3 Table. Diagnostics for Dichotomizing into “High” and “Low” Risk Groups using Different Cutpoints**

| **Cutoint (%)** | **Patients classified as “high risk” (%)** | **Sensitivity (%)**  **Pr(T+\|D+)** | **Specificity (%)**  **Pr(T-\|D-)** | **PPV (%)**  **Pr(D+\|T+)** | **NPV (%)**  **Pr(D-\|T-)** | **LR_positive_**  **P(T+\|D+)/ P(T+\|D-)** | **LR_negative_**  **P(T-\|D+)/ P(T-\|D-)** |
| --- | --- | --- | --- | --- | --- | --- | --- |
| ***Person Level Classification (Primary)*** | | | | | | | |
| 0.0015 | 82.4 | 98.5 | 17.6 | 0.06 | 99.9 | 1.2 | 0.09 |
| 0.002 | 52.5 | 94.1 | 47.5 | 0.09 | 99.9 | 1.8 | 0.12 |
| 0.003 | 32.6 | 87.6 | 67.4 | 0.13 | 99.9 | 2.7 | 0.18 |
| 0.0035 | 23.8 | 83.2 | 76.2 | 0.17 | 99.9 | 3.5 | 0.22 |
| 0.004 | 20.0 | 80.2 | 80.1 | 0.21 | 99.9 | 4.0 | 0.25 |
| 0.005 | 13.5 | 74.4 | 86.5 | 0.27 | 99.9 | 5.5 | 0.30 |
| 0.0075 | 7.9 | 64.4 | 92.2 | 0.40 | 99.9 | 8.3 | 0.39 |
| 0.01 | 5.6 | 58.6 | 94.5 | 0.52 | 99.9 | 10.7 | 0.44 |
| 0.02 | 2.6 | 44.4 | 97.5 | 0.86 | 99.9 | 17.8 | 0.57 |
| 0.03 | 1.7 | 36.8 | 98.4 | 1.09 | 99.9 | 23.0 | 0.64 |
| 0.05 | 1.0 | 27.9 | 99.0 | 1.42 | 99.9 | 27.9 | 0.73 |
| 0.1 | 0.5 | 16.2 | 99.5 | 1.70 | 99.9 | 32.4 | 0.84 |
| 0.15 | 0.2 | 13.1 | 99.8 | 2.13 | 99.9 | 43.9 | 0.87 |
| ***Person-Month Level Classification*** | | | | | | | |
| 0.001 | 84.9 | 97.9 | 15.2 | 0.003 | 99.9 | 1.2 | 0.14 |
| 0.0015 | 44.0 | 93.3 | 56.0 | 0.01 | 99.9 | 2.1 | 0.12 |
| 0.002 | 30.4 | 88.9 | 69.6 | 0.01 | 99.9 | 2.9 | 0.16 |
| 0.0025 | 18.4 | 81.7 | 81.6 | 0.01 | 99.9 | 4.4 | 0.22 |
| 0.003 | 13.9 | 76.8 | 86.1 | 0.01 | 99.9 | 5.5 | 0.27 |
| 0.004 | 8.4 | 69.0 | 91.6 | 0.02 | 99.9 | 8.2 | 0.34 |
| 0.007 | 3.8 | 56.2 | 96.2 | 0.04 | 99.9 | 14.7 | 0.46 |
| 0.01 | 2.4 | 45.3 | 97.6 | 0.05 | 99.9 | 18.9 | 0.56 |
| 0.02 | 1.0 | 30.7 | 99.0 | 0.08 | 99.9 | 30.7 | 0.70 |
| 0.03 | 0.6 | 24.6 | 99.4 | 0.11 | 99.9 | 41.0 | 0.76 |
| 0.05 | 0.3 | 17.4 | 99.7 | 0.15 | 99.9 | 58.0 | 0.83 |
| 0.1 | 0.1 | 10.1 | 99.9 | 0.21 | 99.9 | 79.8 | 0.90 |
| 0.15 | 0.07 | 7.32 | 99.9 | 0.26 | 99.9 | >1000 | 0.93 |
