# Supplementary material for: Predicting overdose among individuals prescribed opioids using routinely collected healthcare utilization data
Source: PLoS One. 2020 Oct 20;15(10):e0241083. doi: 10.1371/journal.pone.0241083 (PMC7575098; doi:10.1371/journal.pone.0241083)
Supplement: S4 Table — (DOCX) [file pone.0241083.s005.docx]

**S4 Table. Example Calculation for the Predicted Probability of Opioid Overdose in the Next Month**

Consider a patient with the following values for each predictor of opioid overdose:

| **Predictor** | **Coefficient** | **Value for Characteristic** |
| --- | --- | --- |
| Intercept | -11.50 |  |
|  |  |  |
| *Categorical Variables* |  |  |
| Age |  |  |
| 18-25 years | 0.79 | 1 |
| 26+ years | ref | ref |
| Opioid dependence | 1.15 | 1 |
| Opioid abuse without dependence | 0.97 | 1 |
| Back and neck pain | 0.14 | 1 |
| Neuropathic pain and fibromyalgia | 0.07 | 0 |
| Chronic pancreatitis | 0.01 | 0 |
| Abdominal pain | 0.04 | 0 |
| Other pain | 0.26 | 1 |
| Cocaine use | 0.15 | 0 |
| Alcohol abuse | 0.59 | 1 |
| Tobacco use | 0.25 | 1 |
| Other substance use | 0.95 | 0 |
| Depression | 0.40 | 0 |
| Bipolar disorder | 0.14 | 0 |
| Psychosis/schizophrenia | 0.56 | 0 |
| Anxiety disorder | 0.16 | 1 |
| Other psychiatric disorders | 0.25 | 0 |
| Suicide attempt | 1.30 | 0 |
| Hepatic disease | 0.14 | 0 |
|  |  |  |
| *Continuous Variables* |  |  |
| Number of extended-release opioid prescriptions dispensed | 0.09 | 5 |
| Total days supplied for all opioid prescriptions dispensed, for 30-days supplied | 0.09 | 3 |
| Total dose (per 1000mg in oral morphine equivalents) for opioid prescriptions dispensed | 0.0001 | 20 |
| Number of unique prescribers for opioids | 0.10 | 1 |
| Number of unique pharmacies for opioid dispensings | 0.17 | 3 |
| Total number of non-opioid prescriptions dispensed | 0.002 | 1 |
| Number of hospitalizations | 0.15 | 0 |
| Number of unique providers seen | 0.01 | 2 |
| Number of urine drug screens | 0.04 | 0 |
| **Predictor** | **Coefficient** | **Value for Characteristic** |
| *Continuous Variables* |  |  |
| Number of opioid dispensings |  |  |
| Fentanyl | 0.13 | 2 |
| Hydrocodone | 0.10 | 0 |
| Hydromorphone | 0.18 | 0 |
| Methadone | 0.15 | 1 |
| Morphine | 0.13 | 5 |
| Oxycodone | 0.14 | 0 |
| Number of non-opioid dispensings |  |  |
| Antidepressants | 0.04 | 0 |
| Benzodiazepines | 0.17 | 1 |
| Gabapentanoids | 0.11 | 0 |
| Muscle relaxants | 0.09 | 0 |
| Other hypnotics | 0.05 | 0 |
|  |  |  |
| *Quadratic Transformations* |  |  |
| Total opioid dispensings | -0.01 | 8 |

The patient’s predicted probability of opioid overdose in the next month would be estimated as follows:

$$\frac{e^{-11.5+0.79+1.15+0.97+0.14+0.26+0.59+0.25+0.16+\left( 0.09*5 \right)+\left( 3*0.09 \right)+\left( 0.0001*20 \right)+\ldots+\left( 0.11*3 \right)+\left( -0.01*8 \right)}}{1+e^{-11.5+0.79+1.15+0.97+0.14+0.26+0.59+0.25+0.16+\left( 0.09*5 \right)+\left( 3*0.09 \right)+\left( 0.0001*20 \right)+\ldots+\left( 0.11*3 \right)+\left( -0.01*8 \right)}}$$

$=0.0190$* 100 = 1.90% predicted probability of opioid overdose in the next month
